# Polymer-Based Nanomaterials for Pharmaceutical, Biomedical and Environmental Applications—New Trends, Benefits and Future Opportunities

**DOI:** 10.3390/nano16140846

**Published:** 2026-07-10

**Authors:** Joanna Rydz, Marta Musioł, Barbara Zawidlak-Węgrzyńska

**Affiliations:** 1 Centre of Polymer and Carbon Materials, Polish Academy of Sciences, M. Curie-Skłodowska 34, 41-819 Zabrze, Poland; 2 Department of Chemistry, Faculty of Medicine in Zabrze, Academy of Silesia, 40-555 Katowice, Poland; barbara.zawidlak@wst.com.pl

Natural or synthetic polymer-based nanomaterials offer a versatile platform with unique properties that make them promising candidates for various applications, especially in the fields of biomedicine and environmental science. The ability to tailor their properties, such as specific surface area, flexibility, excellent biocompatibility, and, depending on the polymer, tailored (bio)degradation, allows for precise design and customization for specific functions, including drug delivery, imaging, and environmental remediation. These materials have opened up new possibilities for advanced technologies that can address complex challenges in healthcare and environmental sustainability [1].

Recently, polymer-based nanomaterials have gained increasing importance in biomedical, pharmaceutical, and environmental applications. Current research focuses on the rational design, synthesis, surface engineering, and functionalization of polymeric nanostructures, emphasizing how nanoscale control enables advanced physicochemical and biological properties. Recent studies demonstrate the versatility of both synthetic and natural polymer systems, including (bio)degradable nanoparticles, polymer nanocomposites, stimuli-responsive materials, and hybrid organic–inorganic nanostructures. These studies also addresses the fabrication of nanocarriers for targeted drug delivery, controlled-release systems, imaging platforms, and antimicrobial materials, underlining the growing importance of polymeric nanomaterials in nanomedicine and personalized therapies. Furthermore, current studies focus on environmentally oriented applications, such as pollutant adsorption, water purification, and sustainable material development, reflecting the increasing interest in green nanotechnology and circular-material approaches. A recurring theme in recent research is the relationship between structure, morphology, and functionality. The presented studies demonstrate how tailoring particle size, surface chemistry, porosity, interfacial interactions, and hierarchical organization can significantly enhance mechanical, optical, electrical, catalytic, and biological properties. In particular, the incorporation of functional nanofillers, surface-modification strategies, and supramolecular approaches has enabled the development of multifunctional systems with improved performance and application-specific behavior [2,3].

Advanced aramid-based polymers have attracted considerable attention due to their exceptional thermal stability, mechanical strength, and potential for use in demanding industrial applications [4]. Two novel aramid copolymers, 1,3-bis(4-aminophenoxy)benzene (MBAB)-aramid and 1,4-bis(4-aminophenoxy)benzene (PBAB)-aramid, were synthesized by incorporating flexible bis(4-aminophenoxy)benzene moieties into the polymer backbone, enabling high-molar-mass polymerization and the fabrication of ultra-thin, transparent films with excellent processability. The obtained materials exhibited outstanding thermal stability and mechanical performance, making them promising candidates for applications in thin films, membranes, electrical insulation, and advanced functional coatings [5].

Polymer-based nanocarriers have emerged as promising platforms for improving the delivery efficiency and therapeutic performance of photosensitizers used in photodynamic therapy [6]. Encapsulation of hypocrellin B, a naturally occurring photosensitizer derived from the traditional Chinese medicinal fungus *Hypocrella bambusae*, into liposomes and poly(lactic-*co*-glycolic acid) (PLGA) nanoparticles preserved its photophysical properties while influencing cellular uptake and intracellular distribution. Both nanoformulations exhibited strong reactive oxygen species generation and effective photodynamic anticancer activity upon laser irradiation [7].

Environmental pollution caused by heavy metal contamination and industrial solid waste accumulation has become a major global challenge, increasing the demand for sustainable wastewater treatment technologies and waste valorization strategies [8]. A fully coal gangue-based SAPO-5 molecular sieve was successfully synthesized through a hydrothermal process and demonstrated excellent adsorption performance toward Cd^2+^ and Pb^2+^ ions, high stability during repeated adsorption cycles, and significant potential for environmentally friendly wastewater remediation applications [9].

Polymer-based composite membranes have gained increasing attention for wastewater treatment due to their potential to improve permeability, hydrophilicity, and antifouling performance [10]. The incorporation of poly(vinyl pyrrolidone) (PVP)ylated-TiO_2_ nanoparticles into poly(vinylidene fluoride) membranes effectively enhanced nanoparticle dispersion and membrane stability, resulting in improved hydrophilicity, water permeability, and antifouling properties, demonstrating strong potential for advanced wastewater filtration applications [11].

The increasing accumulation of micro- and nanoplastics generated from polymer-based materials has become a major environmental concern due to their adverse effects on ecosystems and human health [12]. A polypropylene/clay nanocomposite was investigated under simulated photo-oxidation and mechanical fragmentation conditions, revealing that the incorporation of nanoclay reduced microplastic generation by mitigating polymer degradation processes [13].

Overall, the presented studies clearly demonstrate the rapidly expanding scope and interdisciplinary nature of polymer-based nanomaterials research. The reported advances demonstrate how precise control over polymer composition, morphology, and interfacial interactions enables the development of high-performance materials for biomedical, pharmaceutical, industrial, and environmental applications. In particular, the integration of functional nanofillers, advanced nanocarriers, hybrid nanostructures, and sustainable material-design strategies has opened new opportunities for improving therapeutic efficiency, membrane technologies, environmental remediation, and the reduction of polymer-related pollution. Collectively, these findings emphasize the strong potential of polymer-based nanomaterials to address current technological and environmental challenges while supporting the development of innovative, sustainable, and application-oriented materials.

The aim of this Special Issue is to present the broad range of applications and utilization of polymer-based nanomaterials in pharmaceutical, biomedical, and environmental fields, with a particular focus on recent advances that highlight the potential of these materials in the above-mentioned areas.

## Data Availability

Not applicable.
